# A Retrospective Observational Study on Admissions Timing in a Psychiatry Hospital: Impact of Late-Afternoon Peaks on Patients and Staff

**DOI:** 10.1192/bjo.2025.10468

**Published:** 2025-06-20

**Authors:** Aalap Asurlekar

**Affiliations:** NHS Lanarkshire, Glasgow, United Kingdom

## Abstract

**Aims:** This study investigates the timing and patterns of patient admissions to a community psychiatry hospital, with the goal of optimizing resource allocation by identifying peak arrival times.

**Methods:** This study was conducted at Udston Hospital within NHS Lanarkshire. The hospital comprises two older adult wards, each with a capacity of 20 beds. Patients admitted to these wards were mainly aged over 65 and were admitted either informally or under the Mental Health Act. A duty doctor handles admissions during working hours, 9 am to 5 pm, while an off-site duty doctor covers evenings and weekends.

Data from 50 randomly selected patients admitted between January 2024 and January 2025 were collected using the electronic patient record platform MORSE. The primary outcome was patient arrival time, categorized into predefined time slots. The secondary outcome analysed admission sources (home, care home, or hospital) and whether patients were admitted informally or under detention (Emergency Detention, Short-Term Detention, or Community Treatment Order).

Categorical data analysis was employed to identify any significant trends in admissions.

**Results:** The study identified a notable peak in the afternoon. A majority of admissions, 37 patients (74%), occurred after 2 pm, with 23 patients (46%) being admitted between 2 pm and 4 pm. In contrast, only 7 patients (14%) were admitted between 9 am and 12:59 pm, highlighting an underutilization of morning hours for patient transfers. Half of these admissions were informal and originated from patients’ homes.

**Conclusion:** Late afternoon admissions delay lab results, requiring follow-up by the off-site duty doctor, which may postpone treatment or escalation to the out-of-hours GP. This disruption can affect sleep, a modifiable risk factor for delirium, raising fall risk and worsening outcomes.

Staff are also impacted, particularly during the evening shift and night shift, where reduced resources and increased workloads heighten admission errors, contributing to moral distress and lower job satisfaction.

Systemically, late admissions disrupt patient flow and worsen inefficiencies. Research links evening and weekend admissions to poorer outcomes.

Addressing this issue requires streamlining workflows through measures such as designated admission timeframes for informal patients, prioritizing safer morning hours for non-urgent cases, and optimizing resource allocation through greater staffing levels during peak periods.

These strategies will enhance patient safety, alleviate the strain on staff, and improve overall operational efficiency.

